# Supplementary material for: Development and prospective implementation of a large language model based system for early sepsis prediction
Source: NPJ Digit Med. 2025 May 17;8:290. doi: 10.1038/s41746-025-01689-w (PMC12084535; doi:10.1038/s41746-025-01689-w)
Supplement: Supplementary file 1 — Supplementary Material [file 41746_2025_1689_MOESM1_ESM.pdf]

Supplementary Material for COMPOSER-LLM: Development and Prospective Implementation of a Large Language Model-based System for Early Prediction of Sepsis

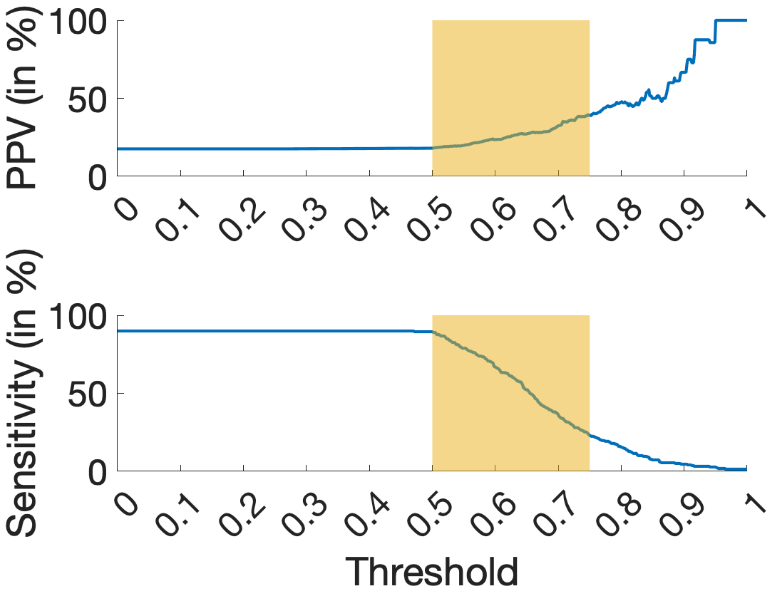

Supplementary Figure 1. Plot of Sensitivity and Positive Predictive Value (PPV) for various decision thresholds. The shaded region represents the uncertainty region in which the positive predictive value is low and near constant and could benefit from using the sepsis likelihood tool to improve sepsis diagnostic certainty.

Supplementary Table 1: Performance on retrospective development cohort

	Sensitivity	PPV	F-1 Score	FAPH
**COMPOSER**	76.7%	27.2%	40.1%	0.036
**COMPOSER-LLM$_{SLT}$ (with sepsis likelihood tool)**	76.5%	33.7%	46.8%	0.024
**COMPOSER-LLM$_{DDx}$ (with differential diagnosis tool)**	72.8%	57.3%	64.1%	0.008

Supplementary Table 2: Outputs generated by the LLM for various clinical signs and symptoms for a patient

	**LLM output**
**Fever**	['No', "Patient's temperature is within normal limits: 98.4 deg F (36.9 deg C)"]; ['No', "Patient's temperature is within normal limits: 98.4 deg F (36.9 deg C)"]; ['No', "Patient's temperature is within normal limits: 98.4 deg F (36.9 deg C)"]
**Hypotension**	['Yes', "Patient's blood pressure is decreased: 73/40"]; ['Yes', "Patient's blood pressure is decreased: 73/40"]; ['Yes', "Patient's blood pressure is decreased: 73/40"]
**Tachypnea**	['No', "Patient's respiratory rate is within normal limits: 20"]; ['No', "Patient's respiratory rate is within normal limits: 20"]; ['No', "Patient's respiratory rate is within normal limits: 20"]
**Tachycardia**	['No', "Patient's pulse is within normal limits: 67"]; ['No', "Patient's pulse is within normal limits: 67"]; ['No', "Patient's pulse is within normal limits: 67"]
**Altered mental status**	['No', 'Patient is alert and oriented.']; ['No', 'Patient is alert and oriented.']; ['No', "No evidence of altered mental status in the patient's history or physical exam."]
**Elevated inflammatory markers**	['Yes', 'White cell count (WBC) elevated to 13.3.']; ['Yes', 'White cell count 13.3, potentially indicative of inflammation.']; ['Yes', 'White cell count is 13.3, which is above the normal range (4.5-11.0 x10^3/ŒºL), indicating inflammation.']
**Positive blood culture**	['No', 'No information about blood culture in the note.']; ['No', 'No information about blood culture in the note.']; ['No', 'No information about blood culture results is available in the medical note.']
**Suspicion of bacterial infection**	['Yes', 'Elevated WBC and suspected UTI based on symptoms and lab results']; ['Yes', 'Elevated WBC and suspected UTI based on symptoms and lab results']; ['Yes', 'Elevated WBC and suspected UTI based on symptoms and urinalysis results']
**Organ dysfunction**	['Yes', 'Decreased blood pressure, abnormal Creatinine level, abnormal WBC count suggestive of renal and hematologic dysfunction.']; ['Yes', 'Decreased blood pressure, abnormal Creatinine level, abnormal WBC count suggestive of renal and cardiovascular dysfunction.']; ['Yes', 'Decreased blood pressure, abnormal Creatinine level, abnormal WBC count suggestive of renal and hematologic dysfunction.']

Supplementary Table 3: COMPOSER-LLM$_{DDx}$ performance on the retrospective validation cohort for various primary decision thresholds (θ_1) at a fixed secondary decision threshold (θ_2) of 0.5. Note that the LLM pipeline was run for samples with $\theta_2 \leq$ sepsis risk scores $< \theta_1$.

Risk score interval for running LLM-based differential diagnosis tool	Sensitivity	Positive predictive value	F1-score
[0.5 - 0.6] (θ_2=0.5, θ_1 = 0.6)	82.5%	24.9%	38.3%
[0.5 - 0.65] (θ_2=0.5, θ_1 = 0.65)	78.4%	30.6%	44.0%
[0.5 - 0.7] (θ_2=0.5, θ_1 = 0.7)	73.9%	38.4%	50.5%
[0.5 - 0.75] (θ_2=0.5, θ_1 = 0.75)	72.1%	52.9%	61.0%
[0.5 - 0.8] (θ_2=0.5, θ_1 = 0.8)	65.1%	64.5%	64.8%

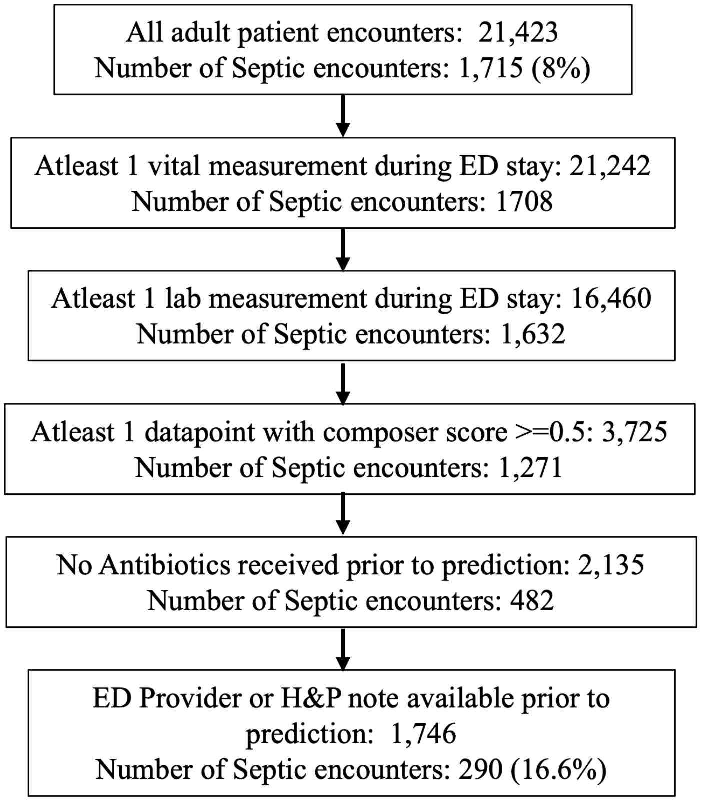

Supplementary Figure 2: Waterfall diagram for the retrospective cohort. Derivation of the final cohort for study analysis after applying exclusion criteria.

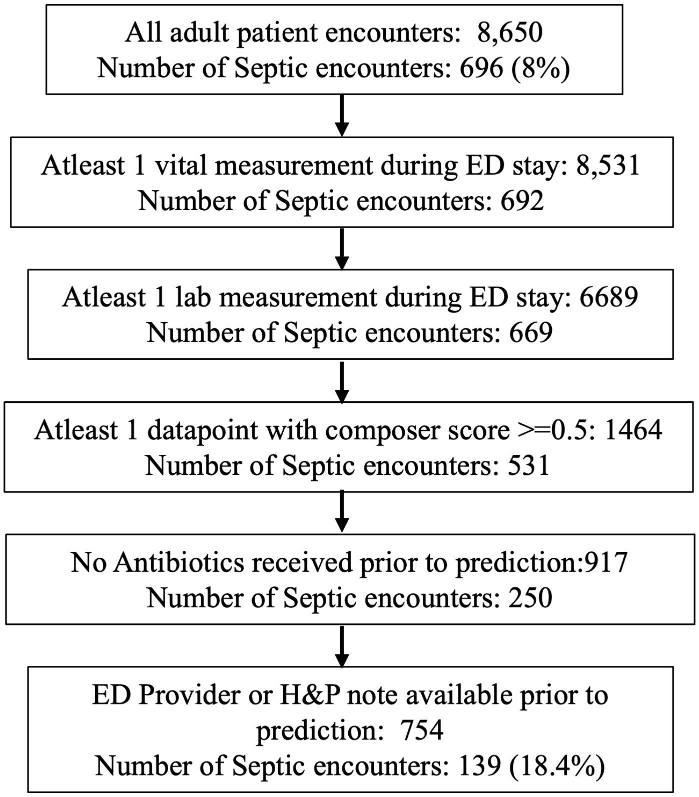

Supplementary Figure 3: Waterfall diagram for the prospective cohort. Derivation of the final cohort for study analysis after applying exclusion criteria.

Supplementary Note 1:

To minimize hallucinations and to maintain consistency in text generation from the LLM, we used a very low temperature (0.3) for the LLM. The temperature parameter controls the level of randomness in the model's output. A lower temperature makes the model's output more deterministic and repetitive. Additionally, for each clinical sign or symptom, the LLM pipeline was run three times and the majority outcome (clinical sign or symptom present or not) across the multiple runs was used for downstream tasks. Finally, two co-authors of the study (J.C.A and G.W.) were asked to perform manual chart review on a sample of 50 patients, to verify the factualness of the LLM outputs. We did not observe significant hallucinations during our chart-reviews. This is likely due to the fact that we do not invoke LLMs with complex reasoning tasks, but rather utilize LLMs for information retrieval based on fairly simple queries for clinical signs and symptoms.
